# Embodied Conversational Agent Appearance for Health Assessment of Older Adults: Explorative Study

**DOI:** 10.2196/19987

**Published:** 2020-09-04

**Authors:** Silke ter Stal, Marijke Broekhuis, Lex van Velsen, Hermie Hermens, Monique Tabak

**Affiliations:** 1 eHealth Group Roessingh Research and Development Enschede Netherlands; 2 Biomedical Systems and Signals Group Faculty of Electrical Engineering, Mathematics and Computer Science University of Twente Enschede Netherlands

**Keywords:** embodied conversational agent, appearance design, health status assessment, older adults, eHealth

## Abstract

**Background:**

Embodied conversational agents (ECAs) have great potential for health apps but are rarely investigated as part of such apps. To promote the uptake of health apps, we need to understand how the design of ECAs can influence the preferences, motivation, and behavior of users.

**Objective:**

This is one of the first studies that investigates how the appearance of an ECA implemented within a health app affects users’ likeliness of following agent advice, their perception of agent characteristics, and their feeling of rapport. In addition, we assessed usability and intention to use.

**Methods:**

The ECA was implemented within a frailty assessment app in which three health questionnaires were translated into agent dialogues. In a within-subject experiment, questionnaire dialogues were randomly offered by a young female agent or an older male agent. Participants were asked to think aloud during interaction. Afterward, they rated the likeliness of following the agent’s advice, agent characteristics, rapport, usability, and intention to use and participated in a semistructured interview.

**Results:**

A total of 20 older adults (72.2 [SD 3.5] years) participated. The older male agent was perceived as more authoritative than the young female agent (*P*=.03), but no other differences were found. The app scored high on usability (median 6.1) and intention to use (median 6.0). Participants indicated they did not see an added value of the agent to the health app.

**Conclusions:**

Agent age and gender little influence users’ impressions after short interaction but remain important at first glance to lower the threshold to interact with the agent. Thus, it is important to take the design of ECAs into account when implementing them into health apps.

## Introduction

As people get older, they are likely to experience frailty, a decline in functional and cognitive abilities such as walking speed, balance control, and working memory [1,2]. Through electronic health (eHealth), frailty can be assessed using digital questionnaires. A large population can be targeted, including those who are less mobile and face difficulties in seeing a caregiver to perform frailty assessment. In addition, digital frailty assessments can be performed on a regular basis, be dynamically adapted based on information provided by the user, and provide immediate results. An eHealth app can coach the user in a personalized way toward a healthy lifestyle based on the outcomes of the frailty assessment. Research shows that collecting health data using a digital survey does not affect test reliability with respect to a paper version [3-5], and several studies showed similar results for a population of older adults [6,7]. In addition, Fanning and McAuley [7] showed that older adults may accept a tablet for health surveys and van Velsen et al [6] showed that older adults preferred a tablet survey to a paper survey.

Research shows that the older and more frail adults get, the more they become nonrespondents to questionnaires [8,9], whereas refusal of face-to-face interviewing is less present in this population [8]. To overcome the problem of lack of face-to-face interaction in a digital frailty assessment, an embodied conversational agent (ECA) can provide an alternative. ECAs are more or less autonomous and intelligent software entities with an embodiment used to communicate with the user [10]. By interacting with the user face to face, ECAs can build trust and rapport—a close and harmonious relationship—leading to companionship and long-term continual use [11].

To establish trust and rapport with the agent, users should have a positive impression of the agent. These impressions can be shaped by static [12] and dynamic characteristics [12,13]. Static characteristics mostly relate to an agent’s visual appearance, often tested using the so-called zero acquaintance approach, where a person observes the agent without interacting with the agent. Dynamic characteristics include an agent’s verbal and nonverbal behaviors and are often tested using a thin-slicing approach, where a person draws inferences about an agent’s personality based on short excerpts of social behavior [14].

Although ECAs have the potential to be used as eHealth apps such as digital frailty assessments, little is known about how these agents should be designed and how the design affects our impressions of the agents, and no design guidelines exist [15]. In one study, ter Stal et al [16] identified people’s first impressions of agents varying in age, gender, and role using a zero acquaintance approach: there was no interaction involved, and participants rated static agent images at first glance. The study shows that characteristics of older and male agents were perceived differently than characteristics of young and female agents, respectively. In addition, older adults seem to prefer a young female over an older male agent. Other research focused on users’ perceptions of static agent images at first glance [17-19], showing that the agent’s gender and role affect the user’s perception of the agent. However, little research exists on people’s impressions after short interactions with agents and how the design of the agents affects these impressions. Therefore, research is needed to investigate how the design of an agent affects users’ impressions of the agent during and after actual interaction (using a thin-slicing approach).

The aim of this study is to assess how an agent’s appearance, particularly age and gender, affects the users’ likeliness of following agent advice and users’ perceptions of the agent’s characteristics and feeling of rapport after short interaction with the agent. This study builds on previous work [16] by studying users’ impressions of agents at first glance (using the zero acquaintance approach) and after a short interaction with the agents (using the thin-slicing approach). As a secondary aim, we investigate the potential of a frailty assessment app with an agent by evaluating its usability and intention to use.

## Methods

### Frailty Assessment App

The ECA under study was embedded within a frailty assessment web app developed as part of a larger platform designed to counter frailty by offering older adults training modules in the domains of healthy nutrition and physical and cognitive training to maintain a healthy lifestyle [20]. Initial and continued use of the platform is stimulated by integrating gamification elements. In this study, we focused on the stand-alone frailty assessment app.

**Figure 1:**
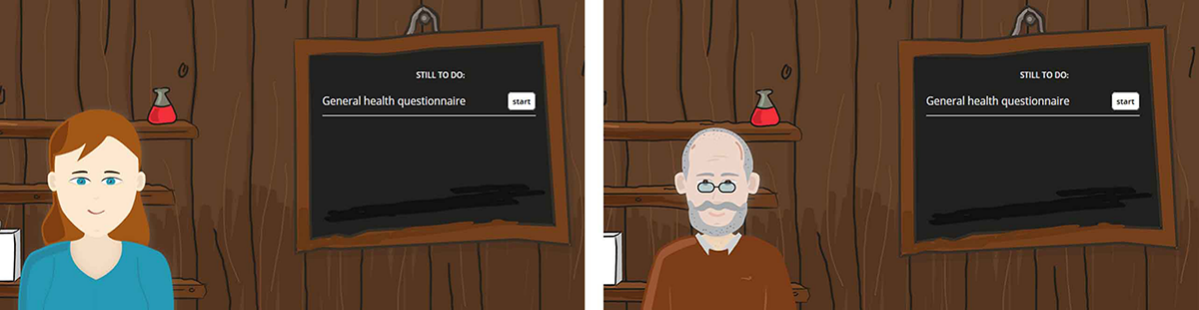
Frailty assessment app: opening page introducing agents Sylvia and Egbert.

**Figure 2:**
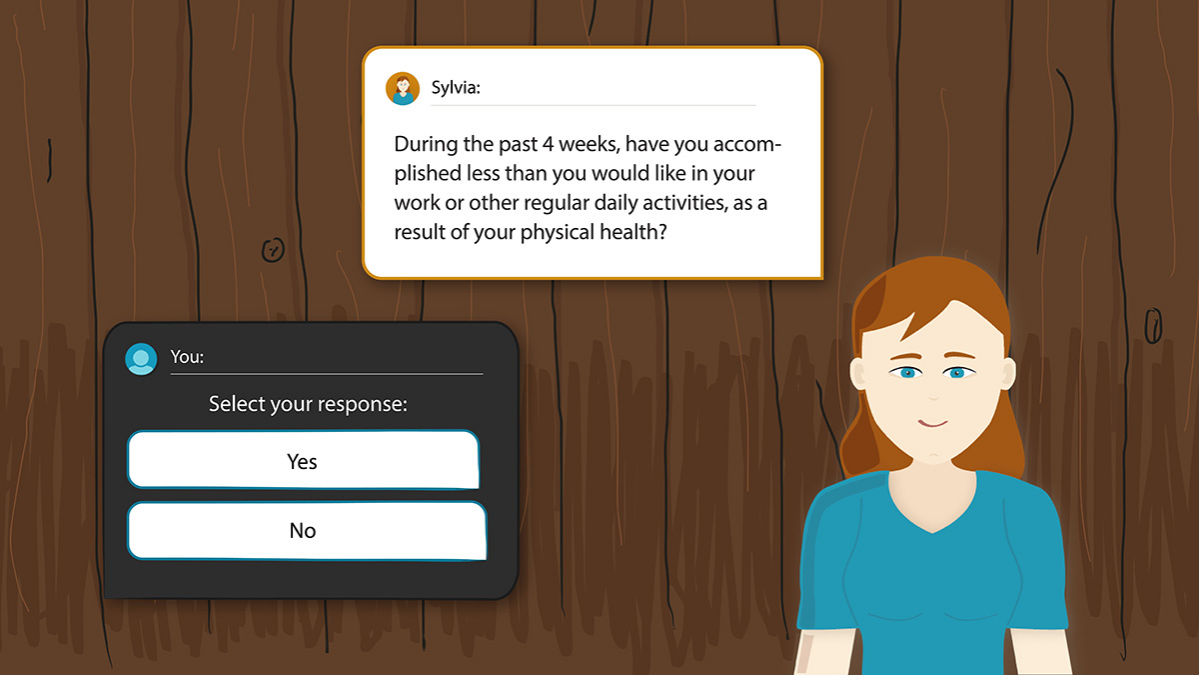
Dialog page with peer agent Sylvia.

The frailty assessment app consisted of an index page (Figure 1) and a dialogue page (Figure 2). On the index page, an agent was displayed next to a blackboard. The blackboard provided a list of available dialogues: introductory small talk, questionnaire assessing aspects of the older adult’s health, and small talk explaining the results of the questionnaires. When a dialogue was finished, the user returned to the index page. Before the questionnaire dialogues were performed, only the introductory small talk was available on the blackboard. In this dialogue, users were introduced to the agent and the goal of the frailty assessment. Afterward, the questionnaire dialogues were unlocked and shown on the blackboard. Three validated questionnaires were implemented to assess the older adult’s frailty status covering multiple health domains. The 36-item Short-Form Health Survey [21] contains 36 multiple-choice questions related to health topics (eg, physical functioning, social functioning). The Alzheimer Disease Detection [22] tests for functional decline in memory using 8 yes or no items. The Mini Nutritional Assessment [23] tests for malnutrition with 6 multiple-choice questions related to nutrition and weight. We translated the three frailty assessment questionnaires into dialogues between the agent and older adults. After questionnaires were completed, the result dialogue was unlocked on the blackboard. In this dialogue, users received the outcomes of the assessment.

Only one dialogue was available at a time. Clicking on the start button of a dialogue opened the dialogue page (Figure 2). A dialogue consisted of multiple dialogue steps. Each dialogue step consisted of a statement by the agent and one or more reply options that could be selected by the user. The statement by the agent was shown in the white box with the orange border and the reply options for the user were listed in the black box. After finishing a dialogue with the agent, the user returned to the index page and available dialogues listed on the blackboard were updated.

**Figure 3:**
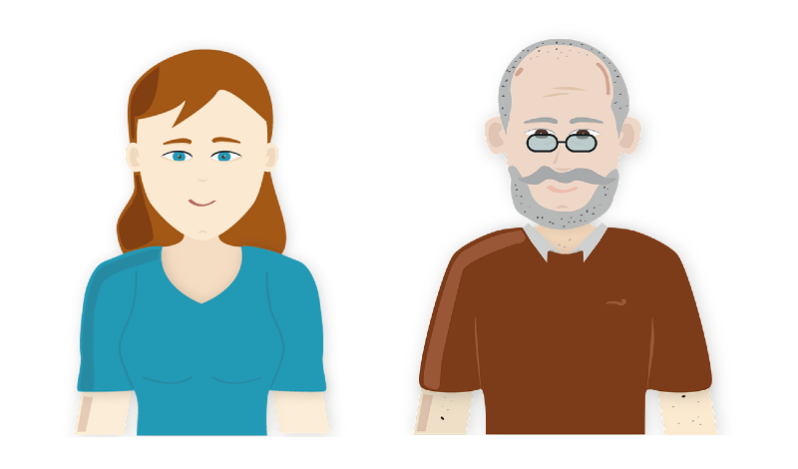
Agents used during the experiment.

The agents used in the frailty assessment app (Figure 3) are Sylvia, a young female peer agent, and Egbert, an older male peer agent. By a peer agent, we mean an agent who is not a medical expert. Agent designs were selected based on findings from a previous study [16], in which the static images of eight agents were evaluated. The agent images differed on three features: age (young or old), gender (male or female), and role (experts had a high level of health expertise, and peers had a low level of health expertise). In an online questionnaire, images of all agents were shown to the participant at once, with participant selecting agent they preferred most (to be their health coach) at first glance. Afterward, participant rated characteristics for each agent. Results showed that a young female agent was preferred most and an older male agent was preferred least in both a general and elderly population (ie, these designs were extremes in terms of user preference). This study builds on the previous study by evaluating users’ impressions of these two agents, both at first glance and after a short interaction with the agents. A blinking eyes animation was implemented for both agents. In addition, when the agent spoke (ie, when a new dialogue step was loaded), a mouth animation of a fixed duration was played.

### Study Design

**Figure 4:**
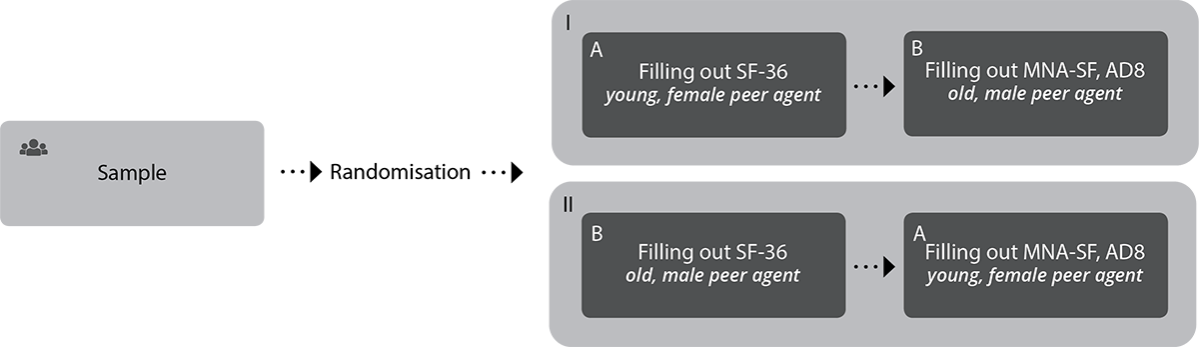
Study design including randomization process.

We applied a within-subject design in which we counterbalanced the order in which agents were presented to participants. Half of the participants started the frailty assessment with the young, female peer agent and finished with the older, male peer agent (Figure 4, top). The other half of the participants were first presented with the older male peer agent, followed by the young, female peer agent (Figure 4, bottom). The study was performed in a lab setting, taking place either at a research institute or a local physiotherapy practice. The nature of this general study with healthy volunteers from the general population does not require formal medical ethical approval according to Dutch law. All participants provided their informed consent.

### Participants

Participants should be aged 65 years or above and fluent in the Dutch language in order to be included. In addition, they should be cognitively able to work with an ECA as assessed via the Mini-Mental State Examination, scoring at least 23 out of 30 points [24]. We recruited the respondents via a Dutch panel of adults that indicated they were interested in participating in research on eHealth. Participants were also recruited via a local physiotherapy practice.

### Measurements

#### Questionnaires

Before interacting with the frailty assessment app, the participant completed the preinteraction questionnaire gathering the participant’s gender, date of birth, education, housing status, technology literacy, health literacy, and state of change for nutrition and physical activity [25].

After interacting with each agent (Figure 4), the participant completed the postinteraction questionnaire. To investigate the effect of the agent’s appearance, we assessed the following:

- Likeliness of following the agent’s advice (on a 7-point Likert scale)
- Agent characteristics ratings (all on 7-point Likert scales): friendliness, authority, involvement, reliability, intelligence
- Agent rapport scale rating (all on 7-point Likert scales) by Acosta and Ward [26]: emotional rapport, cognitive rapport, helpfulness, trustworthiness, likeability, naturalness, enjoyableness, human-likeness, persuasiveness, recommendability

Secondarily, we investigated the usability of the frailty assessment app and the intention to use the frailty assessment app on a single 7-point Likert scale.

#### Thinking Aloud

In order for us to triangulate the quantitative data, participants were asked to think aloud while interacting with the frailty assessment app. Audio was recorded and screen captures were taken. The researcher did not help or support the participant but only reminded the participant to think out loud when necessary.

#### Interviews

At the end of the session, the participant was interviewed. The interview was semistructured and guided by asking the user’s opinion regarding positive and negative aspects around the effect of the agent’s appearance, usability of the frailty assessment app, and intention to use the frailty assessment app.

### Data Analyses

SPSS Statistics 25 (IBM Corporation) software was used to perform statistical analyses. Since the underlying data were nonparametric, for all relations testing differences between the two agents, a Wilcoxon signed-rank test was conducted. All tests used a 95% confidence interval. All variables were tested for statistically significant differences between the two agents by means of a model consisting of Wilcoxon signed-rank tests for cross-over designs. Effect size was calculated by *r*=Z/√N, using 0.1, 0.3, and 0.5 as cutoff values for a small, medium, and large effects, respectively.

The audio recordings of the thinking aloud sessions and interviews were transcribed and inductively thematically analyzed. In addition, screen captures of the interaction with the frailty assessment app were aligned with the audio recordings. This way, the screen captures were used to verify the thoughts of the participants on the audio recordings. All themes were coded using ATLAS.ti 8 (ATLAS.ti Scientific Software Development GmbH) based on an empirical method proposed by Pope and Mays [27]. One researcher (StS) created a first coding scheme based on the data and then labeled the transcripts. A second researcher (MB) used the coding scheme to code a subset of the data so that a discussion could be held between the first and second coder for improving the coding scheme. The procedure of creating a first coding scheme, labeling the data by two researchers, and discussing the coding scheme was repeated a second time leading to a final coding scheme. The final coding scheme was used by the first coder to code all data for final analyses. The final coding scheme contained the following codes: agent characteristics, appearance agents, interaction with agents, preference agent, content questionnaires, language usage in dialogues, presentation information, interaction with app, design, navigation, general computer interaction, and intention to use.

## Results

### Participants

A total of 21 participants began the study (Table 1). One participant was not able to complete the protocol due to a lack of computer experience and was excluded. The average age of participants was 72.2 (SD 3.5) years, and 13 males and 7 females participated. Ten participants started with the young, female agent, and ten participants started with the older, male agent.

**Table 1:** Participant demographics (n=20).

Demographic		Value, n (%)
**Education**		
	Elementary school	1 (5)
	High school	1 (5)
	Vocational education	8 (40)
	College	6 (30)
	University	4 (20)
**Living situation**		
	Living alone	1 (5)
	Living with a partner	19 (95)
**Stage-of-change nutrition**		
	Maintenance	18 (90)
	Precontemplation	2 (10)
**Stage-of-change physical activity**		
	Maintenance	13 (65)
	Action	3 (15)
	Contemplation	1 (5)
	Precontemplation	2 (20)
	Unknown	1 (5)
**Technology literacy level**		
	Moderate or high	20 (100)
**Health literacy level**		
	Moderate or high	19 (95)
	Low	1 (5)
**Physical limitations**		
	No risk of facing physical limitations	9 (45)
	Risk of facing physical limitations	10 (50)
	Already faced physical limitations	1 (5)
**Cognitive limitations (Mini-Mental State Examination)**		
	No risk of facing cognitive limitations (score ≥23)	19 (95)
	Risk of facing cognitive limitations (score <23)	1 (5)

### Agent Appearance

#### Ratings Questionnaire

**Figure 5:**
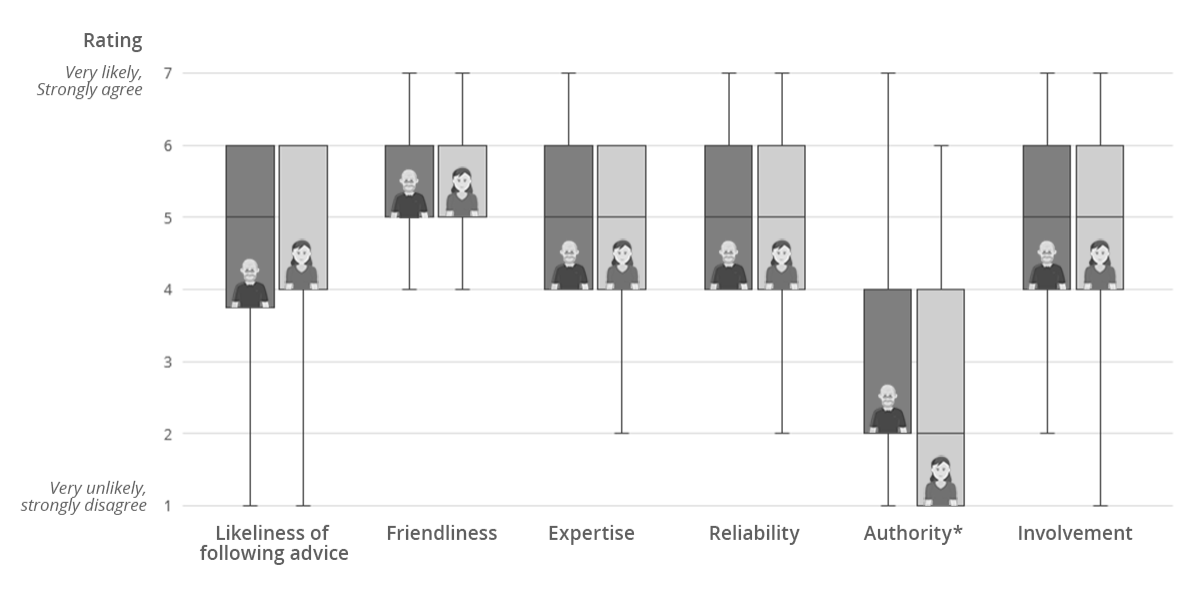
Ratings of the likeliness of following advice and characteristics of the two agents (*P*<.05).

**Figure 6:**
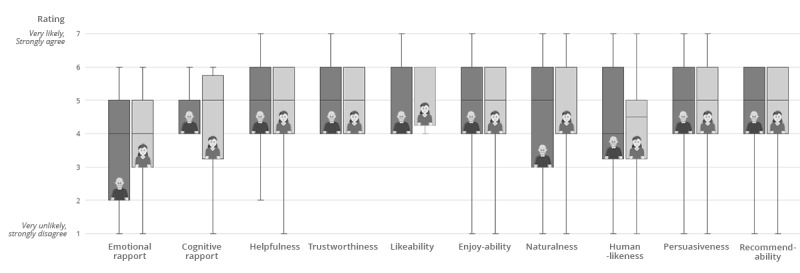
Ratings of the rapport scale items of the two agents.

Table 2 shows the questionnaire results regarding (1) the likeliness of following the agent’s advice, (2) users’ perceptions of the agent characteristics (eg, friendliness, expertise), and (3) users’ feeling of rapport (eg, emotional rapport, helpfulness) for both agents. Corresponding box plots can be seen in Figure 5 and Figure 6. For the ratings of the likeliness of following the agent’s advice, no significant difference between Egbert and Sylvia was found. However, Egbert was rated significantly more authoritative than Sylvia (*P*=.03), resulting in a medium effect size (*r*=.344). No significant differences were found between the agents for all other agent characteristics and the rapport scale items.

Analysis of the thinking aloud sessions and interviews resulted in the following themes on the effects of agent appearance: agent characteristics, agent appearances, interaction with the agents, and agent preferences.

**Table 2:** Results of the Wilcoxon signed-rank tests (n=19 or 20) comparing the mean ranks of the ratings of likeliness of following the agent’s advice, agent characteristics, and rapport scale items.

Characteristic		Median Egbert (Q1-Q3)	Median Sylvia (Q1-Q3)	*z* score	*P* value
Likeliness of following advice		5.0 (3.3-6.0)	6.0 (4.0-6.0)	–1.613	.11
**Agent characteristics**					
	Friendliness	6.0 (5.0-6.0)	6.0 (5.0-6.0)	–0.264	.79
	Expertise	5.0 (4.0-6.0)	5.0 (4.0-6.0)	–0.966	.33
	Reliability	5.0 (4.0-6.0)	5.0 (4.0-6.0)	–0.276	.78
	Authority	2.0 (2.0-4.0)	2.0 (1.0-4.0)	–2.121	*.03
	Involvement	4.5 (4.0-6.0)	5.0 (4.0-6.0)	–0.158	.88
**Rapport scale**					
	Emotional rapport	4.0 (2.0-5.0)	4.0 (3.0-5.0)	–1.310	.19
	Cognitive rapport	4.0 (4.0-5.0)	5.0 (3.3-5.8)	–0.829	.41
	Helpfulness	5.0 (4.0-6.0)	5.0 (4.0-6.0)	–0.877	.38
	Trustworthiness	5.0 (4.0-6.0)	5.0 (4.0-6.0)	0	>.99
	Likeability	6.0 (4.0-6.0)	6.0 (4.3-6.0)	–0.604	.55
	Naturalness	5.0 (4.0-6.0)	5.0 (4.0-6.0)	–0.491	.62
	Enjoyability	5.0 (3.0-6.0)	4.0 (4.0-6.0)	–0.182	.86
	Human-likeness	4.0 (3.3-6.0)	4.5 (3.3-5.0)	–0.486	.63
	Persuasiveness	5.0 (4.0-6.0)	5.0 (4.0-6.0)	–0.942	.35
	Recommendability	5.0 (4.0-6.0)	5.0 (4.0-6.0)	–0.368	.71

#### Users’ Perceptions of Agent Characteristics

A few participants indicated they had trouble getting an impression of the agents’ personalities or found it difficult to connect personality to ECAs in general. A few others perceived the agents as natural and not artificial. On the other hand, the majority did not perceive the agents as human: they perceived the agents as cartoons, static dolls, computers, or machines.

It is a computer, it is still interaction from a distance, it does not become personal, it does not have any personality, I do not feel a connection.

Male, 68 years

The agents remain computers, you cannot call them friendly or unfriendly, they are computers and I do not connect any human characteristics to them.

Male, 78 years

In the interviews, some participants indicated they did not perceive the agents differently with respect to their personality. A few participants explained that both agents used friendly language, whereas others argued the agents were friendly, since they responded in a way that fit the situation and provided compliments. In addition, a participant explained that both agents were not too young or too old and seemed to be modern people due to responses such as “Gosh, how nice.” Also, this participant said he liked that the agents were not too young, since a young agent would not have much experience. One participant particularly indicated that the female agent was friendlier than the male agent, whereas another participant believed that the male agent was more highly educated and more intellectual than the female agent.

#### Users’ Perceptions of Agent Appearances

A participant indicated that the agents looked like cartoons or drawings, whereas she preferred the agents to look like real humans. This participant also indicated that the blinking eyes and mouth animation were distracting.

The rest of the comments related to the appearance of either one of the agents. One person particularly mentioned the female agent having a friendly face, whereas all other comments related to the male agent. The appearance of the older male agent evoked several associations, such as the agent looking old, and, therefore, unhealthy. Others associated the older male agent with a scientific staff member, a nerd or a male of the type of wearing sandals with socks, because of his glasses and popular beard. Participants preferred an energetic, spontaneous person and one that is more neutral and clean-shaven. One participant did not like the male agent, because he associated the agent with his or her uncle, having a similar name: a spoiled man with whom you would not be able to connect. Another participant found the male agent more distracting than the female agent, because of his glasses.

#### Users’ Perceptions of Interaction With the Agents

Several participants explicitly indicated that they expected or would like the agent to speak. One participant expected the agent to speak due to its mouth animation, whereas another had this expectation, since humans interact via speech in real conversations. Another participant pointed out that, due to the absence of agent speech, the user has to multitask: the user simultaneously has to read and answer the questions and pay attention to the agent. Therefore, she would like the agent to speak.

Well, I have to read what you say to me, but instead open your mouth yourself!

Female, 73 years

Other opinions on the interaction with the agent focused on the naturalness of the interaction.

It felt as if there was a real human in front of me.

Female, 71 years

Another participant described the interaction as actually talking to someone, and yet another participant described the interaction as having a phone call, in which someone is checking how you are doing. Some participants were less positive. A few participants specifically said that the interaction with the agents was impersonal.

Actually, I do not have the feeling I am really communication with someone.

Female, 65 years

Another participant said that she did not take part in a conversation but was simply reading and answering questions. This participant did not establish a connection with the agents.

I barely know her.

Female, 65 years

Understanding each other? Then one would expect interaction.

Female, 65 years

Last, some comments related to the implemented small talk. On the one hand, some participants seemed to like the small talk, reflected by them laughing. On the other hand, a participant was irritated by the implemented small talk, she felt being treated like a child.

#### Agent Preference

The majority of the participants indicated they did not prefer one agent over the other. Most of them indicated they did not have a preference, since they perceived the agents to be similar. Some did not even remember they interacted with two different agents. However, some participants did show a preference. Most participants preferred the female agent, either because they believed she was friendlier or discussed a more interesting topic. Only one participant preferred the male agent but could not say why.

### Usability and Intention to Use Frailty Assessment App

Questionnaire results show that the usability of and intention to use the frailty assessment app were high: the 20 usability ratings displayed a median of 6.1 (interquartile range [IQR] 6.1-7.0) and the 20 intention-to-use ratings displayed a median of 6.0 (IQR 4.0-6.0) on a 7-point Likert scale.

During the thinking aloud session and interviews, participants pointed out usability issues of the frailty assessment app or provided suggestions for improvements to the app. The following themes were identified: content questionnaires (mentioned 107 times), language usage in dialogues (mentioned 41 times), presentation information (mentioned 21 times), interaction with app (mentioned 14 times), design (mentioned 7 times), navigation (mentioned 7 times), and general computer interaction (mentioned 6 times).

Most comments or suggested improvements related to the content of the questionnaires and the language in the app. The majority of the participants reported that the questionnaires did not fit their personal situation and contained a lot of repetition or ambiguity. Participants suggested adapting the questionnaires according to previous answers given. In addition, participants commented on the language used: words being ambiguous, too popular or too old fashioned, unnecessary, patronizing, or not being known by people with a lower education or older adults. Furthermore, participants commented on the length and structure of the sentences and pointed out spelling mistakes. A participant suggested adapting the language in the app to the education of the user. Considerably fewer comments related to the presentation of information, interaction with the app, design or navigation of the app, and general computer interaction. As an example, with respect to navigation, some participants indicated they would like to be able to go back to a previous dialogue step.

With respect to the intention to use, the thinking aloud sessions and interviews showed that a minority of the participants would like to use the app. A participant indicated he would not use the app but would recommend the app to others who might benefit from it. In addition, some participants clearly indicated they would not use the app. The majority of the participants indicated that the agents did not add any benefit to the app, arguing that the app was not personal since answer options were limited and the opportunity to explain them was missing. A participant stated that for the app to be beneficial, it should also provide advice on what actions the user should perform to become more healthy. Another participant explicitly stated that he would use the app when the text was replaced by speech.

## Discussion

### Principal Findings

Our results show that the appearance of an agent, in particular age and gender, affects users’ perceptions of agent authority but does not affect users’ perceptions of other agent characteristics, users’ feelings of rapport, or users’ likeliness of following agent advice. Compared with a young female agent, an older male agent is only seen as more authoritative. These results are not in line with our expectation that agents are perceived differently after a short interaction with a user. To the best of our knowledge, there is no existing research comparing users’ impressions of agents at first glance with those after short interactions. But research shows that in human-human interaction, first impressions, formed within milliseconds [28], are difficult to lose. Therefore, we assumed that the differences in perceptions of characteristics of a static image of a young female agent and an older male agent, as found in a previous study [16], would still be present after a short interaction with these agents. An explanation for this inconsistency could be that impressions in human-agent interaction differ from impressions in human-human interaction. Users’ judgments of agents may modify with ongoing interaction, as research shows that agents do have a second chance to make a first impression [13,29]. Therefore, differences in perceptions of both agents may have been present at first glance but disappeared after interaction. Further research is needed to confirm this finding. Future research could study users’ perceptions of agent characteristics with a larger study population. Eventually agents will be used in a long-term setting; therefore, it is interesting to research not only users’ perceptions at first glance and after short-term interaction, but also after long-term interaction.

How do we explain the difference in perceptions of agent authority after a short interaction? Although research on short-term interaction with an agent indicates that an agent’s appearance, including clothing [18], racial concordance with the user [30,31], and similarity with the user [30,32], could affect users’ perceptions of the agent, to the best of our knowledge there is no research on agent authority after short interaction in particular. From a previous study [16], we see that at first glance, static images of male and older agents are indeed seen as more authoritative than female and young agents, respectively. In addition, the study shows that the differences found in authority are often higher compared with differences found for other characteristics tested, which could explain why the difference in authority level is still present after short interaction. However, since we did not control the age and gender of the agents in this study independently, it is difficult to say whether the difference in perception of agent authority is caused by agent age or gender in particular or solely by the combination. Future research could study which factors actually control the difference, researching users’ perceptions of agent authority by independently controlling the age and gender of the agents. In addition, future research could study how an agent’s authority is perceived after long-term interaction.

We expect that the effect of the first impression established by agent age and gender on the impression after short interaction is small compared with the effect of other design features, such as the content and language of the messages, (absence of) agent speech, and the amount of embodiment. Our study shows that the majority of participants perceived the agents not as humans but as machines or cartoons and found interaction with the agents impersonal or artificial. They did not have the feeling of being in a conversation. These perceptions may indicate users had a negative adaptation gap [29], which occurs when a user overestimates the competency of an agent, creating a negative gap between expected and actual competency of the agent and resulting in the user being disappointed. This negative adaptation gap may have been caused by the content and language of agent messages, agents lacking speech, or agents having little embodiment, as supported by remarks made by participants during the thinking aloud sessions and interviews. Therefore, we believe it is important to manage users’ expectations of agent characteristics and functionality up front, ensuring users’ expectations match actual agent capabilities by explaining what the users can expect from the agent. Future research could study how an agent’s content, language, speech, and embodiment affect users’ perceptions of the conversation with the agent (eg, how these factors could make the conversation with an agent more human-like).

Although our study shows agent age and gender have little effect on users’ impressions of the agent after short interaction, we believe that adapting these features to the user is important because they affect users’ impressions of the agent at first glance [17,19,33], and research shows that people with favorable impressions of someone tend to interact more with that person than they do others who gave unfavorable impressions [34]. Selecting an agent with the right age and gender could thus lower the threshold to interact with the agent and use the app.

Second, our results show that usability of the developed frailty assessment app was judged positively overall; issues identified by participants related to the content or language of the questionnaires. We suggest tailoring the content and language toward the personal characteristics of the user, as confirmed by existing research [35], and adapting the content to previous answers given by the user.

Third, not all participants show an intention to use the app. Research indicates that older adults put effort into learning new digital technologies as long as they are believed to be worthy of time and dedication (eg, when technology can be used to keep in touch with others to foster relationships [36]). Similarly, research shows that the elderly value apps that address a social problem [37]. The app used in our study did not address a social problem, which could have resulted in some participants not seeing the added value of the app and not showing an intention to use the app. In addition, intention to use digital technologies in elderly persons is, next to the quality of the technology itself, affected by their personal context (eg, their ability to concentrate) and social context (eg, whether family is around to provide technical support) [37]. Both factors might have affected participant intentions to use the frailty assessment app in our study.

More specifically, the majority of participants do not believe the agent adds value to the frailty assessment app. Therefore, we suggest updating the design of the agent. We believe that the agent should convey additional information to its message in text via its embodiment. Existing research provides evidence for implementation of animations of the agent’s embodiment, showing that animations positively affect users’ impressions of the agent [38-40] and interaction time [13,39]. In addition, the use of speech is recommended because it could increase the sense of personality of an agent [41] and could be used to describe feelings [42]. Low-literate users could benefit from multiple output modalities [43]. Furthermore, participants indicated they would like the app to provide advice on what actions they should perform in order to become more healthy. We see an opportunity for using the agent to provide this advice. As an example, the agent could show videos of exercises to improve physical strength.

### Strengths and Limitations

This is the first study that specifically evaluates effects of agent appearance after short interaction with the agent. In addition, this study uses actual health content, which is scarce in research on agent design.

Our study also has some limitations. First, the negative adaptation gap between user expectations of agent capabilities and actual agent capabilities suggests the app used might not have been mature enough. The agent conveyed the majority of the information via text. Participants might have been focused on reading the text and therefore paid little attention to the agent, resulting in participants having difficulties in creating impressions of agent characteristics and establishing rapport. Second, interaction time with the agents might have been too short to create impressions of agent characteristics and establish rapport. Third, although we found a difference in users’ perceptions of authority of the young female and the older male ECA, it is difficult to identify whether this was caused by the ECA’s gender or age, since these factors were not independently controlled in the study.

### Toward Digital Frailty Assessment With Embodied Conversational Agents: Recommendations for Future Research

#### Agent Design Implications

First, convey empathy or emotion using the agent’s embodiment. This way, agent design can positively affect users’ impressions of the agent and interaction time. Second, reduce the user’s cognitive load by providing the agent messages in speech. This way, agent design can positively affect users’ impressions of the agent. Third, select an agent appearance that fits the age and gender of the user. This way, agent design can lower the threshold to start using the app.

#### Prerequisites Frailty Assessment

First, take into account the user’s personal situation, such as disabilities and living situation, and adapt the content. Adapt the questionnaire so users do not see questions that do not apply to their situation. Second, save the answers given by the user, and adapt the questionnaire accordingly. This way, users do not have to answer questions that are not applicable to them. Third, adapt the agent’s language based on the educational level of user so the language is neither too simple nor too complex.

### Conclusions

Our study shows that an agent’s appearance, in particular age and gender, only affects users’ perceptions of agent authority after short-term interaction. We conclude that adapting agent age and gender to users’ preferences is important to lower the threshold to interact, whereas the content and language of the agent’s messages and agent speech and embodiment are important factors for users’ impressions of the agent after short interaction.

We believe that ECAs have potential to be used in digital frailty assessment, but future research is needed. Future research could study users’ perceptions of agents after long-term interaction, whether users’ perceptions of agent authority are related to agent age or gender in particular, and how an agent’s content, language, speech, and embodiment affect users’ perceptions of the conversation with the agent.

## Abbreviations

ECA: embodied conversational agent
eHealth: electronic health
IQR: interquartile range

## References

[1] Malva JO, Bousquet J (2016). Operational definition of active and healthy ageing: Roadmap from concept to change of management. Maturitas.

[2] Fried LP, Tangen CM, Walston J, Newman AB, Hirsch C, Gottdiener J, Seeman T, Tracy R, Kop WJ, Burke G, McBurnie MA (2001). Frailty in older adults: evidence for a phenotype. J Gerontol A Biol Sci Med Sci.

[3] Bliven BD, Kaufman SE, Spertus JA (2001). Electronic collection of health-related quality of life data: validity, time benefits, and patient preference. Qual Life Res.

[4] Kvien TK, Mowinckel P, Heiberg T, Dammann KL, Dale O, Aanerud GJ, Alme TN, Uhlig T (2005). Performance of health status measures with a pen based personal digital assistant. Ann Rheum Dis.

[5] Hess R, Santucci A, McTigue K, Fischer G, Kapoor W (2008). Patient difficulty using tablet computers to screen in primary care. J Gen Intern Med.

[6] van Velsen L, Frazer S, N'dja A, Ammour N, Del Signore S, Zia G, Hermens H (2018). The reliability of using tablet technology for screening the health of older adults. Stud Health Technol Inform.

[7] Fanning J, McAuley E (2014). A comparison of tablet computer and paper-based questionnaires in healthy aging research. JMIR Res Protoc.

[8] Hébert R, Bravo G, Korner-Bitensky N, Voyer L (1996). Refusal and information bias associated with postal questionnaires and face-to-face interviews in very elderly subjects. J Clin Epidemiol.

[9] Hardie JA, Bakke PS, Mørkve O (2003). Non-response bias in a postal questionnaire survey on respiratory health in the old and very old. Scand J Public Health.

[10] Ruttkay Z, Dormann C, Noot H, Ruttkay Z, Pelachaud C (2004). Embodied conversational agents on a common ground: a framework for designevaluation. From Brows to Trust: Evaluating Embodied Conversational Agents.

[11] Vardoulakis L, Ring L, Barry B, Sidner C, Bickmore T (2012). Designing relational agents as long term social companions for older adults. Proc 12th Int Conf Intell Virt Agents.

[12] Cafaro A, Vilhjálmsson HH, Bickmore T (2016). First impressions in human-agent virtual encounters. ACM Trans Comput-Hum Interact.

[13] Bergmann K, Eyssel F, Kopp S (2012). A second chance to make a first impression? How appearance and nonverbal behavior affect perceived warmth and competence of virtual agents over time. International Conference on Intelligent Virtual Agents.

[14] Vartanian O, Stewart K, Mandel DR, Pavlovic N, McLellan L, Taylor PJ (2012). Personality assessment and behavioral prediction at first impression. Personality and Individual Differences.

[15] ter Stal S, Kramer LL, Tabak M, op den Akker H, Hermens H (2020). Design features of embodied conversational agents in eHealth: a literature review. Int J Hum-Comput Stud.

[16] ter Stal S, Tabak M, op den Akker H, Beinema T, Hermens H (2019). Who do you prefer? The effect of age, gender and role on users’ first impressions of embodied conversational agents in eHealth. Int J Hum–Comput Interact.

[17] Forlizzi J, Zimmerman J, Mancuso V, Kwak S (2007). How interface agents affect interaction between humans and computers. Proc on 2007 Con Designing Pleasurable Products Interfaces.

[18] Parmar D, Olafsson S, Utami D, Bickmore T (2018). Looking the part: the effect of attire and setting on perceptions of a virtual health counselor. Proc Int Conf Intell Virtual Agents.

[19] Zimmerman J, Ayoob E, Forlizzi J, McQuaid M (2005). Putting a face on embodied interface agents.

[20] Noorman-de Vette F (2019). Designing Game-Based eHealth Applications Strategies for Sustainable Engagement of Older Adults [Dissertation].

[21] van der Zee K, Sanderman R (1993). Het meten van de algemene gezondheidstoestand met de rand-36.

[22] Galvin JE, Roe CM, Coats MA, Morris JC (2007). Patient's rating of cognitive ability: using the AD8, a brief informant interview, as a self-rating tool to detect dementia. Arch Neurol.

[23] Rubenstein LZ, Harker JO, Salvà A, Guigoz Y, Vellas B (2001). Screening for undernutrition in geriatric practice: developing the short-form mini-nutritional assessment (MNA-SF)). J Gerontol A Biol Sci Med Sci.

[24] Kok R, Verhey F (2002). Gestandaardiseerde Mini-Mental State Examination.

[25] Prochaska JO, Velicer WF (1997). The transtheoretical model of health behavior change. Am J Health Promot.

[26] Acosta JC, Ward NG (2011). Achieving rapport with turn-by-turn, user-responsive emotional coloring. Speech Commun.

[27] Mays N, Pope C (1995). Qualitative research: observational methods in health care settings. BMJ.

[28] Bar M, Neta M, Linz H (2006). Very first impressions. Emotion.

[29] Komatsu T, Kurosawa R, Yamada S (2011). How does the difference between users’ expectations and perceptions about a robotic agent affect their behavior?. Int J of Soc Robotics.

[30] Zhou S, Bickmore T, Paasche-Orlow M, Jack B (2014). Agent-user concordance and satisfaction with a virtual hospital discharge nurse. Proc Int Conf Intell Virtual Agents.

[31] Zhou S, Zhang Z, Bickmore T (2017). Adapting a persuasive conversational agent for the Chinese culture.

[32] Wissen V, Vinkers C, Halteren A (2016). Developing a virtual coach for chronic patients: a user study on the impact of similarity, familiarity and realism. Proc Int Conf on Pers Technology.

[33] Nguyen H, Masthoff J (2007). Is it me or is it what I say? Source image and persuasion. Proc Int Conf on Pers Technology.

[34] Kelley HH (1950). The warm-cold variable in first impressions of persons. J Pers.

[35] Beukema S, van Velsen L, Jansen-Kosterink S, Karreman J (2017). "There is something we need to tell you…": communicating health-screening results to older adults via the internet. Telemed J E Health.

[36] Lindley S, Harper R, Sellen A (2009). Desiring to be in touch in a changing communications landscape: Attitudes of older adults. Proc SIGCHI Conf Hum Factors Comput Syst.

[37] Waycott J, Vetere F, Pedell S, Morgans A, Ozanne E, Kulik L (2016). Not for me: older adults choosing not to participate in a social isolation intervention. Proc 2016 CHI Con Hum Factors Comput Syst.

[38] Baylor AL, Ryu J (2016). The effects of image and animation in enhancing pedagogical agent persona. J Educ Comput Res.

[39] Kang S, Feng A, Leuski A, Casas D, Shapiro A (2015). The effect of an animated virtual character on mobile chat interactions. Int Conf Hum-Agent Interact.

[40] Cowell A, Stanney K (2003). Embodiment and interaction guidelines for designing credible trustworthy embodied conversational agents. Int Conf Intell Virtual Agents.

[41] Nass C, Lee K (2000). Does computer-generated speech manifest personality? An experimental test of similarity-attraction. Conf Hum Factors Compu Syst.

[42] Veletsianos G, Miller C, Doering A (2009). Enali: a research and design framework for virtual characters and pedagogical agents. J Educ Comput Res.

[43] Thies M (2015). User interface design for low-literate and novice users: past, present and future. Found Trends Hum-Agent Interact.

